# Supplementary material for: Prospect on Rare Earth Elements and Metals Fingerprint for the Geographical Discrimination of Commercial Spanish Wines
Source: Molecules. 2020 Nov 28;25(23):5602. doi: 10.3390/molecules25235602 (PMC7730952; doi:10.3390/molecules25235602)
Supplement: Supplementary file 1 [file molecules-25-05602-s001.pdf]

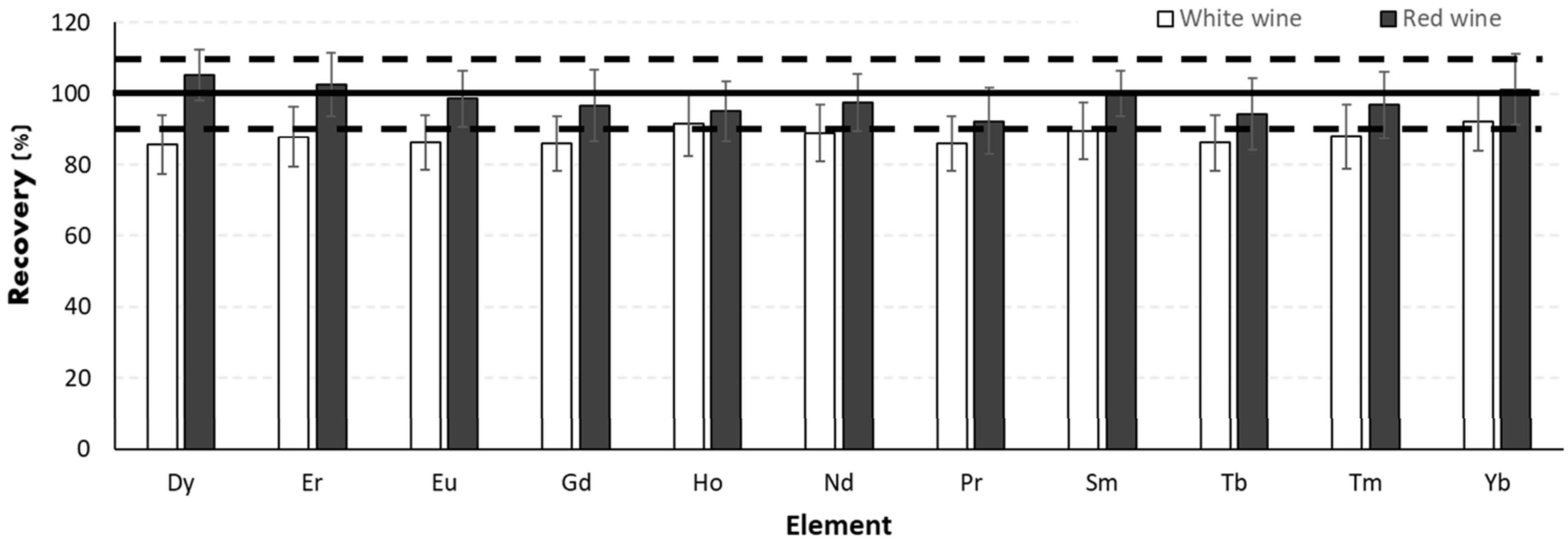

Figure S1. Analytical recoveries with the hTISIS operated at 125 ºC and 30 µL min^{-1} for two real wine samples. Solid line indicates values of 100% for the recovery, whereas the dotted lines indicated 90% and 110% values. Spike concentration: 50 µg kg^{-1}.

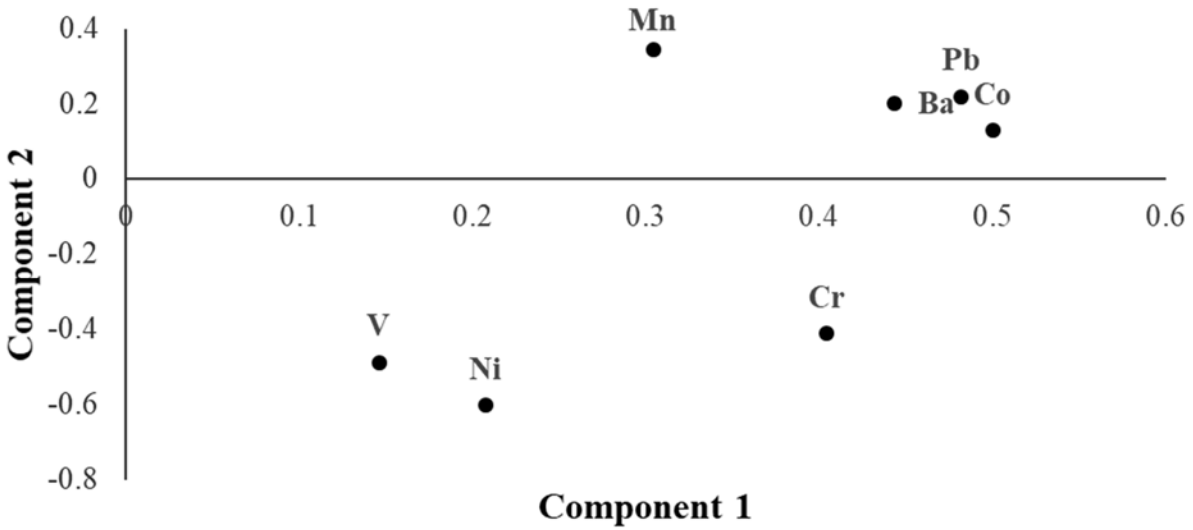
Mn
Pb
Ba
Co
Cr
V
Ni
0.4
0.2
0
-0.2
-0.4
-0.6
-0.8
0
0.1
0.2
0.3
0.4
0.5
0.6
Component 2
Component 1

(a)

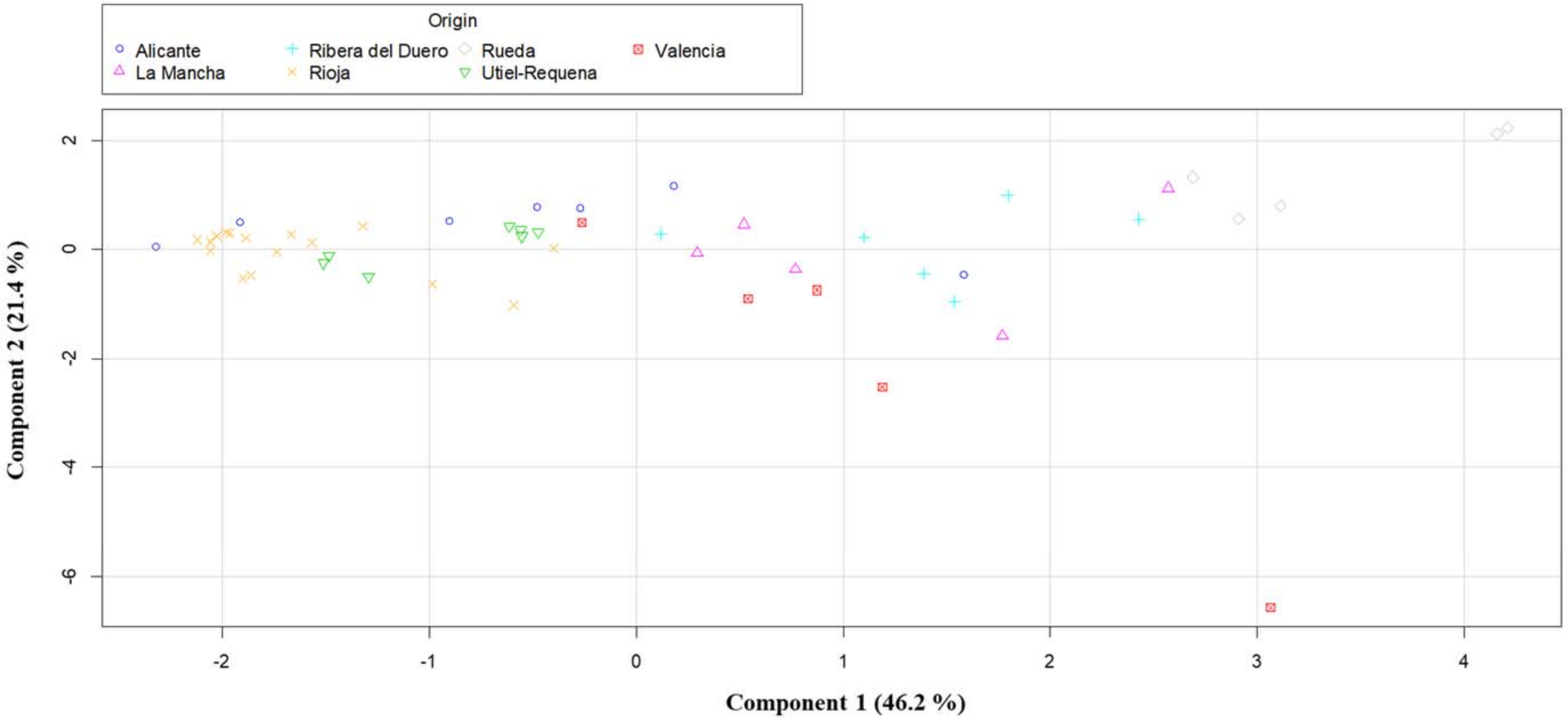

(b)

Figure S2. Principal components analysis performed on data expressed as concentration of elements in wines from different designation of origin. a) loading plot; b) score plot. Elements: Ba, Co, Cr, Mn, Ni, Pb and V.

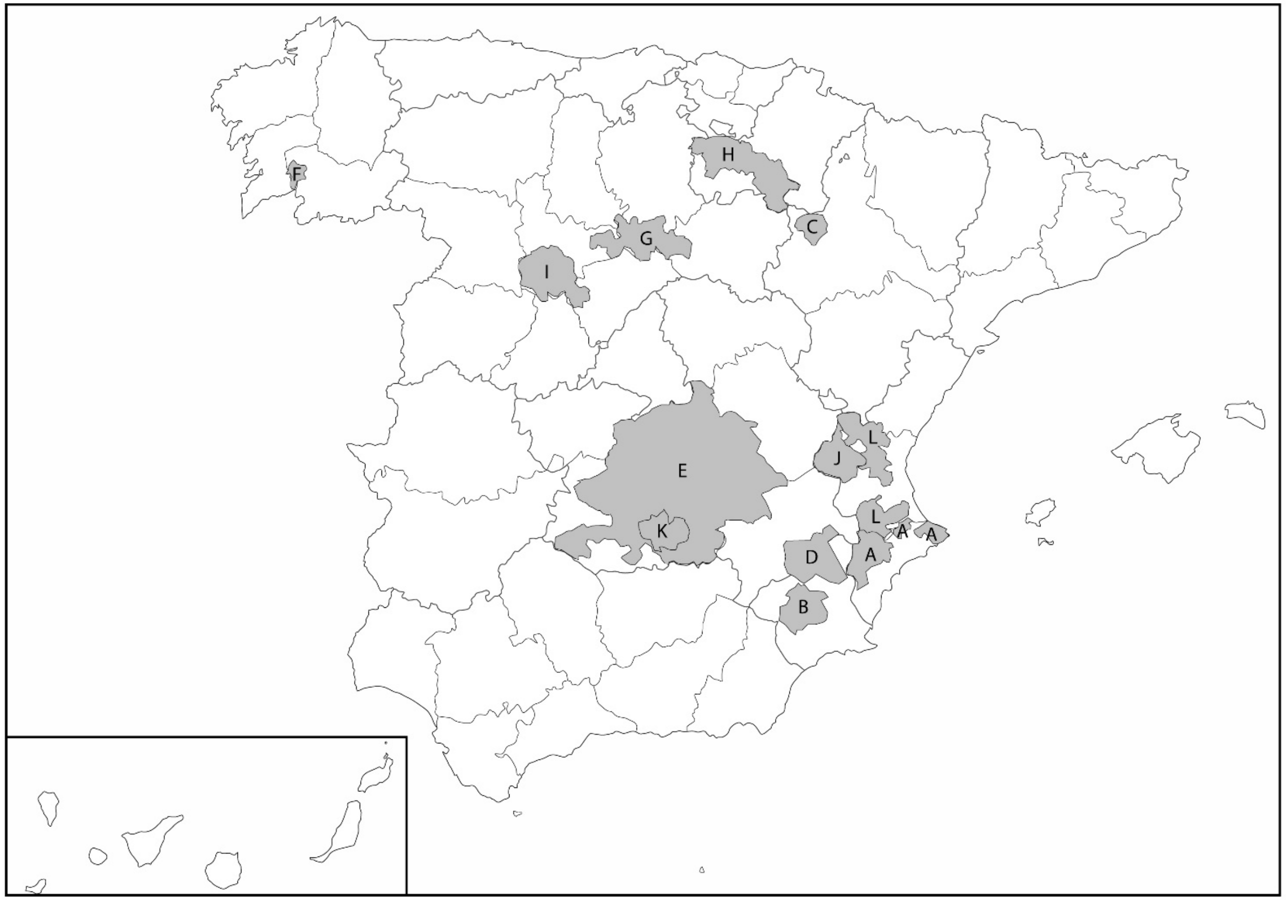

Figure S3. Map of Spain showing locations of the Protected Designation of Origin (PDO) wines evaluated on this study. PDO: (a) Alicante; (b) Bullas; (c) Campo de Borja; (d) Jumilla; (e) Castilla la Mancha; (f) Ribeiro; (g) Ribera del Duero; (h) Rioja; (i) Rueda; (j) Utiel-Requena; (k) Valdepeñas; (l) Valencia.

Table S1. Rare earth elements concentration (μg kg^{-1}) in Rioja wine samples (mean ± 95%-confidence interval).

Sample	Dy	Er	Eu	Gd	Ho	La	Lu	Nd	Pr	Sm	Tb	Tm	Yb
RJ1	1.09 ± 0.06	0.87 ± 0.04	1.32 ± 0.07	3.11 ± 0.17	0.86± 0.03			1.59 ± 0.09	0.83 ± 0.07	0.97 ± 0.07	0.91 ± 0.03	0.75 ± 0.04	1.071 ± 0.011
RJ2	1.03 ± 0.05	0.92 ± 0.06	1.45 ± 0.06	4.4 ± 0.3	0.84 ± 0.04	1.62 ± 0.11	0.100 ± 0.010	1.83 ± 0.08	0.99 ± 0.03	1.08 ± 0.03	0.94 ± 0.05	0.69 ± 0.05	1.07 ± 0.07
RJ3	1.20 ± 0.12	1.06 ± 0.10	1.39 ± 0.05	3.6 ± 0.4	0.95 ± 0.09	0.74 ± 0.06	0.41 ± 0.04	2.3 ± 0.2	1.17 ± 0.03	1.22 ± 0.10	0.99 ± 0.08	0.83 ± 0.07	1.14 ± 0.11
RJ4	0.96 ± 0.08	0.92 ± 0.09	1.26 ± 0.11	3.7 ± 0.4	0.87 ± 0.04	1.26 ± 0.11	0.214 ± 0.018	2.15 ± 0.13	1.01 ± 0.06	1.09 ± 0.10	0.87 ± 0.06	0.68 ± 0.04	0.97 ± 0.07
RJ5	0.98 ± 0.03	0.70 ± 0.04	0.97 ± 0.05	1.75 ± 0.15	0.56 ± 0.02			1.56 ± 0.06	0.46 ±0.04	0.53 ± 0.04	0.69 ± 0.02	0.453 ± 0.016	0.43 ± 0.04
RJ6	1.60 ± 0.06	1.42 ± 0.05	1.576 ± 0.007	2.2 ± 0.2	1.260 ± 0.015	0.25 ± 0.02	0.99 ± 0.06	3.48 ± 0.16	1.66 ± 0.09	1.67 ± 0.13	1.28 ± 0.10	0.78 ± 0.07	1.53 ± 0.14
RJ7	1.72 ± 0.15	1.44 ± 0.13	1.9 ± 0.2	3.8 ± 0.4	1.39 ± 0.12	0.18 ± 0.02	0.56 ± 0.05	2.4 ± 0.3	1.56 ± 0.13	2.0 ± 0.2	1.56 ± 0.14	0.82 ± 0.07	1.67 ± 0.15
RJ8	1.27 ± 0.08	0.97 ± 0.07	1.37 ± 0.07	4.3 ± 0.3	0.69 ± 0.04	1.24 ± 0.11		4.5 ± 0.3	1.39 ± 0.03	1.39 ± 0.11	0.75 ± 0.06	0.55 ± 0.05	1.15 ± 0.11
RJ9	0.96 ± 0.08	0.71 ± 0.06	1.20 ± 0.06	2.65 ± 0.13	0.57 ± 0.03			2.4 ± 0.2	0.91 ± 0.03	0.97 ± 0.10	0.62 ± 0.03	0.43 ± 0.03	0.70 ± 0.06
RJ10	0.97 ± 0.04	0.94 ± 0.03	1.34 ± 0.07	3.31 ± 0.11	0.80 ± 0.03			2.84 ± 0.13	1.09 ± 0.04	1.05 ± 0.05	0.68 ± 0.03	0.46 ± 0.03	0.75 ± 0.04
RJ11	1.74 ± 0.19	1.74 ± 0.15	2.4 ± 0.2	2.6 ± 0.2	1.70 ± 0.16		1.12 ± 0.08	2.08 ± 0.13	1.92 ± 0.14	2.2 ± 0.2	1.99 ± 0.17	1.05 ± 0.09	1.85 ± 0.13
RJ12	1.54 ± 0.13	1.22 ± 0.11	1.04 ± 0.09	1.81 ± 0.11	0.82 ± 0.07			3.7 ± 0.3	1.31 ± 0.11	1.21 ± 0.06	0.81 ± 0.07	0.60 ± 0.05	1.34 ± 0.11
RJ13	1.61 ± 0.11	1.30 ± 0.08	0.93 ± 0.08	1.92 ± 0.13	0.74 ± 0.05			4.59 ± 0.13	1.43 ± 0.08	1.50 ± 0.13	0.66 ± 0.03	0.53 ±0.03	1.44 ± 0.09

RJ14	1.05 ± 0.03	0.84 ± 0.03	1.09 ± 0.01	1.38 ± 0.07	0.80 ± 0.04		1.61 ± 0.06	0.63 ± 0.02	0.69 ± 0.04	0.80 ± 0.02	0.45 ± 0.23	1.06 ± 0.06
RJ15	2.22 ± 0.11	1.48 ± 0.04	1.00 ± 0.05	2.92 ± 0.10	0.75 ± 0.02		5.1 ± 0.3	1.87 ± 0.04	2.25 ± 0.04	0.69 ± 0.05	0.40 ± 0.02	1.37 ± 0.04
RJ16	1.29 ± 0.03	1.06 ± 0.07	0.98 ± 0.07	2.01 ± 0.11	0.69 ± 0.04		4.5 ± 0.3	1.32 ± 0.06	1.27 ± 0.08	0.51 ± 0.03	0.38 ± 0.02	1.40 ± 0.11

Table S2. Rare earth elements concentration (μg kg^{-1}) in Alicante wine samples (mean ± 95%-confidence interval).

Sample	Dy	Er	Eu	Gd	Ho	Lu	Nd	Pr	Sm	Tb	Tm	Yb
A1	1.73 ± 0.08	1.22 ± 0.12	2.03 ± 0.12	1.80 ± 0.11	0.74 ± 0.09		5.8 ± 0.3	1.73 ± 0.08	1.83 ± 0.12	0.70 ± 0.08	0.60 ± 0.04	1.24 ± 0.09
A2	2.5 ± 0.3	2.20 ± 0.11	3.9 ± 0.3	3.3 ± 0.4	1.49 ± 0.15	0.487 ± 0.015	6.5 ± 0.7	2.9 ± 0.2	3.0 ±0.3	1.44 ± 0.17	1.17 ± 0.11	2.8 ± 0.3
A3	3.14 ± 0.16	1.95 ± 0.14	2.03 ± 0.07	3.48 ± 0.11	1.00 ± 0.03		14.9 ± 0.5	3.55 ± 0.09	3.04 ± 0.08	0.95 ± 0.04	0.59 ± 0.06	2.12 ± 0.09
A4	1.83 ± 0.19	1.47 ±0.08	2.19 ± 0.09	1.74 ± 0.17	0.87 ± 0.06		5.46 ± 0.16	1.71 ± 0.11	1.74 ±0.06	0.82 ± 0.10	0.63 ± 0.04	2.22 ± 0.16
A5	1.31 ± 0.10	0.95 ± 0.05	1.71 ± 0.06	1.64 ± 0.10	0.55 ± 0.03		3.5 ± 0.3	1.03 ± 0.04	1.58 ± 0.10	0.50 ± 0.04	0.36 ± 0.03	1.03 ± 0.08
A6	1.45 ± 0.05	1.36 ± 0.05	1.77 ± 0.05	1.75 ± 0.05	1.00 ± 0.03		3.27 ± 0.06	1.34 ± 0.05	1.65 ±0.07	0.90 ± 0.05	0.74 ± 0.04	1.76 ± 0.05
A7	1.70 ± 0.06	1.17 ± 0.06	1.81 ± 0.05	1.95 ± 0.04	0.92 ± 0.03		6.6 ± 0.2	2.01 ± 0.04	2.04 ±0.05	0.92 ± 0.06	0.64 ± 0.02	1.50 ± 0.08

Table S3. Rare earth elements concentration (μg kg^{-1}) in Bullas wine samples (mean ± 95%-confidence interval).

Sample	Dy	Er	Eu	Gd	Ho	Nd	Pr	Sm	Tb	Tm	Yb
B1	0.66 ± 0.06	0.55 ± 0.06	1.66 ± 0.06	0.89 ± 0.06	0.43 ± 0.04	3.71 ± 0.12	0.94 ± 0.05	1.15 ± 0.06	0.41 ± 0.03	0.32 ± 0.03	0.67 ± 0.06
B2	1.02 ± 0.11	0.72 ± 0.08	1.30 ± 0.04	1.37 ± 0.08	0.59 ± 0.05	5.07 ± 0.10	1.52 ± 0.06	1.49 ± 0.11	0.62 ± 0.04	0.43 ± 0.03	0.70 ± 0.07
B3	1.40 ± 0.09	0.80 ± 0.05	1.50 ± 0.10	1.76 ± 0.08	0.55 ± 0.06	7.0 ± 0.2	1.45 ± 0.08	2.09 ± 0.17	0.61 ± 0.02	0.36 ± 0.04	0.72 ± 0.05

Table S4. Rare earth elements concentration (µg kg^{-1}) in Campo de Borja wine samples (mean ± 95%-confidence interval).

Sample	Dy	Er	Eu	Gd	Ho	Nd	Pr	Sm	Tb	Tm	Yb
CB1	0.65 ± 0.05	0.56 ± 0.07	1.01 ± 0.11	1.33 ± 0.08	0.49 ± 0.05	1.61 ± 0.17	0.66 ± 0.05	0.70 ± 0.08	0.47 ± 0.05	0.36 ± 0.04	0.62 ± 0.07
CB2	0.64 ± 0.05	0.56 ± 0.06	1.08 ± 0.06	1.66 ± 0.18	0.53 ± 0.07	1.51 ± 0.17	0.72 ± 0.04	0.85 ± 0.09	0.55 ± 0.06	0.44 ± 0.05	0.64 ± 0.06

Table S5. Rare earth elements concentration (µg kg^{-1}) in Jumilla wine samples (mean ± 95%-confidence interval).

Sample	Dy	Er	Eu	Gd	Ho	Nd	Pr	Sm	Tb	Tm	Yb
J1	1.48 ± 0.06	1.25 ± 0.06	2.08 ± 0.09	2.21 ± 0.18	0.84 ± 0.06	3.2 ± 0.2	1.11 ± 0.09	1.4 ± 0.06	0.97 ± 0.04	0.67 ± 0.05	1.49 ± 0.06
J2	1.9 ± 0.2	1.67 ± 0.19	2.2 ± 0.2	2.8 ± 0.3	1.37 ± 0.14	2.93 ± 0.2	1.76 ± 0.09	2.1 ± 0.2	1.50 ± 0.13	1.03 ± 0.08	1.76 ± 0.09

Table S6. Rare earth elements concentration (µg kg^{-1}) in Castilla la Mancha wine samples (mean ± 95%-confidence interval).

Sample	Dy	Er	Eu	Gd	Ho	Nd	Pr	Sm	Tb	Tm	Yb
M1	1.50 ± 0.06	1.28 ± 0.05	1.66 ± 0.09	2.34 ± 0.03	0.97 ± 0.04	2.89 ± 0.18	1.30 ± 0.07	1.37 ± 0.13	0.86 ± 0.05	0.69 ± 0.03	1.59 ± 0.08
M2	2.9 ± 0.3	2.5 ± 0.3	3.3 ± 0.3	3.7 ± 0.3	1.9 ± 0.2	5.8 ± 0.5	2.3 ± 0.3	2.42 ± 0.11	1.58 ± 0.14	1.24 ± 0.11	3.0 ± 0.2
M3	3.0 ± 0.2	2.44 ± 0.15	2.11 ± 0.19	3.7 ± 0.2	1.09 ± 0.06	13.9 ± 0.8	2.50 ± 0.3	2.78 ± 0.10	0.96 ± 0.05	0.74 ± 0.02	2.6 ± 0.2
M4	1.53 ± 0.06	1.28 ± 0.07	1.97 ± 0.04	1.97 ± 0.07	0.68 ± 0.03	3.9 ± 0.2	1.40 ± 0.08	1.40 ± 0.10	0.73 ± 0.02	0.552 ± 0.010	1.53 ± 0.08
M5	1.86 ± 0.07	1.53 ± 0.06	2.94 ± 0.08	2.61 ± 0.04	0.98 ± 0.03	3.5 ± 0.2	1.32 ± 0.04	1.72 ± 0.09	0.96 ± 0.04	0.71 ± 0.02	1.73 ± 0.07

Table S7. Rare earth elements concentration (µg kg^{-1}) in Ribeiro wine samples (mean ± 95%-confidence interval).

Sample	Dy	Er	Eu	Gd	Ho	Nd	Pr	Sm	Tb	Tm	Yb
R1	2.7 ± 0.3	2.03 ± 0.18	4.0 ± 0.4	6.0 ± 0.6	2.2 ± 0.2	4.1 ± 0.4	2.50 ± 0.11	3.1 ± 0.3	2.29 ± 0.18	2.0 ± 0.2	2.4 ± 0.2
R2	2.00 ± 0.08	1.80 ± 0.15	2.71 ± 0.12	7.4 ± 0.6	1.42 ± 0.08	5.2 ± 0.3	2.21 ± 0.16	2.27 ± 0.11	1.58 ± 0.12	1.29 ± 0.06	2.19 ± 0.16
R3	1.82 ± 0.09	1.60 ± 0.07	4.49 ± 0.14	4.58 ± 0.12	1.33 ± 0.05	4.99 ± 0.19	1.99 ± 0.09	2.3 ± 0.2	1.45 ± 0.04	1.05 ± 0.04	2.03 ± 0.03

Table S8. Rare earth elements concentration (μg kg^{-1}) in Ribera del Duero wine samples (mean ± 95%-confidence interval).

Sample	Dy	Er	Eu	Gd	Ho	Nd	Pr	Sm	Tb	Tm	Yb
RD1	1.42 ± 0.09	1.16 ± 0.08	2.57 ±0.06	2.17 ± 0.15	1.09 ± 0.02	3.1 ± 0.2	1.23 ± 0.07	1.78 ± 0.09	1.11 ± 0.03	0.69 ± 0.04	1.66 ± 0.08
RD2	1.09 ± 0.07	1.10 ± 0.04	1.88 ± 0.07	1.94 ± 0.10	0.93 ± 0.02	4.7 ± 0.3	1.50 ± 0.10	1.56 ± 0.07	1.04 ± 0.02	0.68 ± 0.03	1.50 ± 0.03
RD3	2.6 ± 0.2	2.0 ± 0.2	3.6 ± 0.2	4.0 ± 0.3	1.72 ± 0.14	5.0 ± 0.4	2.8 ± 0.2	2.9 ± 0.2	1.99 ± 0.15	1.23 ± 0.08	1.99 ± 0.12
RD4	9.2 ± 0.3	4.44 ± 0.16	5.37 ± 0.17	11.5 ± 0.3	2.06 ± 0.10	58.9 ± 1.6	14.4 ± 0.3	12.6 ± 0.5	2.29 ± 0.08	0.99 ± 0.04	3.00 ± 0.15
RD5	2.3 ± 0.2	2.1 ± 0.2	3.6 ± 0.3	3.7 ± 0.3	1.9 ± 0.2	3.5 ± 0.3	2.2 ± 0.2	3.0 ± 0.2	2.1 ± 0.2	1.41 ± 0.11	1.99 ± 0.11
RD6	1.58 ± 0.05	1.25 ± 0.04	2.72 ± 0.09	3.97 ± 0.09	1.17 ± 0.03	4.47 ± 0.17	2.07 ± 0.07	1.87 ± 0.06	1.29 ± 0.03	0.94 ± 0.03	1.28 ± 0.03

Table S9. Rare earth elements concentration (μg kg^{-1}) in Rueda wine samples (mean ± 95%-confidence interval).

Sample	Dy	Er	Eu	Gd	Ho	Nd	Pr	Sm	Tb	Tm	Yb
RU1	2.80 ± 0.13	2.18 ± 0.19	2.17 ± 0.15	3.8 ± 0.2	1.45 ± 0.13	4.6 ± 0.3	1.83 ± 0.13	2.2 ± 0.2	1.43 ± 0.13	1.12 ± 0.10	2.39 ± 0.19
RU2	2.11 ± 0.12	2.02 ± 0.16	2.47 ± 0.10	4.7 ± 0.3	1.16 ± 0.06	5.1 ± 0.2	1.68 ± 0.10	1.86 ±0.14	1.09 ± 0.04	0.91 ± 0.04	2.36 ± 0.08
RU3	1.36 ± 0.11	1.60 ± 0.03	2.50 ± 0.09	2.80 ± 0.10	0.902 ± 0.015	1.55 ± 0.14	0.81 ± 0.06	1.11 ± 0.09	0.78 ± 0.04	0.77 ± 0.03	2.92 ± 0.16
RU4	2.12 ± 0.15	2.41 ± 0.13	2.53 ± 0.15	10.3 ± 0.7	1.63 ± 0.13	3.35 ± 0.14	1.68 ± 0.10	1.99 ± 0.10	1.77 ± 0.11	1.58 ± 0.05	3.52 ± 0.17
RU5	1.42 ± 0.06	1.29 ± 0.11	1.98 ± 0.11	3.42 ± 0.19	0.83 ± 0.03	3.75 ± 0.14	1.29 ± 0.05	1.29 ± 0.10	0.80 ± 0.03	0.635 ± 0.014	1.59 ± 0.15

Table S10. Rare earth elements concentration (μg kg^{-1}) in Utiel-Requena wine samples (mean ± 95%-confidence interval).

Sample	Dy	Er	Eu	Gd	Ho	Nd	Pr	Sm	Tb	Tm	Yb
U1	0.93 ± 0.08	0.81 ± 0.04	1.14 ± 0.07	0.87 ± 0.07	0.50 ± 0.04	2.8 ± 0.2	0.85 ± 0.07	0.89 ± 0.07	0.44 ± 0.04	0.32 ± 0.03	0.90 ± 0.08
U2	0.69 ± 0.05	0.37 ± 0.02	0.88 ± 0.08	0.54 ± 0.02	0.40 ± 0.03	0.82 ± 0.04	0.41 ± 0.03	0.52 ± 0.04	0.396 ± 0.018	0.30 ± 0.02	0.38 ± 0.03
U3	0.74 ± 0.07	0.57 ± 0.05	1.23 ± 0.07	1.37 ± 0.11	0.46 ± 0.03	2.07 ± 0.10	0.72 ± 0.05	0.78 ± 0.06	0.47 ± 0.04	0.33 ± 0.03	0.68 ± 0.05
U4	0.60 ± 0.03	0.54 ± 0.04	1.19 ± 0.03	0.63 ± 0.05	0.55 ± 0.03	1.02 ± 0.06	0.58 ± 0.03	0.77 ± 0.06	0.58 ± 0.05	0.45 ± 0.03	0.53 ± 0.04
U5	0.72 ± 0.03	0.39 ± 0.03	1.05 ± 0.07	0.58 ± 0.03	0.36 ± 0.02	1.01 ± 0.05	0.45 ± 0.03	0.54 ± 0.04	0.36 ± 0.03	0.282 ± 0.014	0.43 ± 0.04
U6	0.98 ± 0.05	0.76 ± 0.06	1.20 ± 0.06	0.88 ± 0.07	0.47 ± 0.03	1.51 ± 0.13	0.89 ± 0.03	1.07 ± 0.09	0.42 ± 0.03	0.305 ± 0.012	0.82 ± 0.07
U7	0.96 ± 0.09	0.98 ± 0.08	1.99 ± 0.17	1.40 ± 0.13	0.72 ± 0.07	2.4 ± 0.2	0.96 ± 0.08	1.85 ± 0.16	0.97 ± 0.08	0.84 ± 0.07	0.90 ± 0.08

Table S11. Rare earth elements concentration (μg kg^{-1}) in Valdepeñas wine samples (mean ± 95%-confidence interval).

Sample	Dy	Er	Eu	Gd	Ho	Nd	Pr	Sm	Tb	Tm	Yb
VP1	1.32 ± 0.08	1.07 ± 0.10	2.11 ± 0.06	2.77 ± 0.10	0.80 ± 0.07	2.70 ± 0.14	1.14 ± 0.03	1.33 ± 0.11	0.87 ± 0.06	0.68 ± 0.04	1.16 ± 0.08
VP2	1.48 ± 0.11	1.19 ± 0.08	1.94 ± 0.12	1.83 ± 0.08	0.66 ± 0.05	3.6 ± 0.3	1.14 ± 0.08	1.43 ± 0.13	0.61 ± 0.03	0.453 ± 0.018	1.26 ± 0.11
VP3	1.60 ± 0.07	1.31 ± 0.04	1.51 ± 0.06	2.3 ± 0.2	0.81 ± 0.04	3.70 ± 0.14	1.29 ± 0.08	1.44 ± 0.14	0.77 ± 0.04	0.59 ± 0.05	1.40 ± 0.09
VP4	1.95 ± 0.18	1.58 ± 0.12	2.8 ± 0.2	3.6 ±0.3	1.49 ± 0.12	2.8 ± 0.3	1.68 ± 0.14	2.3 ± 0.2	2.10 ± 0.18	1.30 ± 0.12	1.73 ± 0.15

Table S12. Rare earth elements concentration (μg kg^{-1}) in Valencia wine samples (mean ± 95%-confidence interval).

Sample	Dy	Er	Eu	Gd	Ho	Nd	Pr	Sm	Tb	Tm	Yb
V1	2.41 ± 0.06	1.30 ± 0.03	1.42 ± 0.06	1.62 ± 0.07	1.15 ± 0.02	3.7 ± 0.3	1.56 ± 0.04	1.73 ± 0.04	0.44 ± 0.03	0.55 ± 0.02	2.13 ± 0.08
V2	4.4 ± 0.2	3.4 ± 0.2	1.95 ± 0.08	4.4 ± 0.2	1.31 ± 0.10	16.3 ± 0.5	4.4 ± 0.2	4.0 ± 0.2	1.11 ± 0.08	0.90 ± 0.04	4.56 ± 0.09
V3	3.6 ± 0.4	3.2 ± 0.3	3.5 ± 0.3	4.2 ± 0.3	2.2 ± 0.2	7.7 ± 0.6	3.5 ± 0.3	3.4 ± 0.2	2.3 ± 0.2	1.01 ± 0.05	4.3 ± 0.3
V4	2.5 ± 0.2	2.22 ± 0.12	1.76 ± 0.04	2.79 ± 0.07	1.09 ± 0.06	6.5 ± 0.4	2.13 ± 0.08	2.18 ± 0.10	0.94 ± 0.07	0.84 ± 0.06	3.53 ± 0.13
V5	2.31 ± 0.12	2.20 ± 0.11	1.65 ± 0.06	1.86 ± 0.05	1.15 ± 0.05	4.8 ± 0.2	1.73 ± 0.09	2.10 ± 0.05	1.03 ± 0.05	0.90 ± 0.05	3.23 ± 0.15

Table S13. Analysis of variance of the rare earth elements content in Spanish commercial wines (n=50, F (95%) = 2.31).

	Dy	*Er*	*Eu*	*Gd*	*Ho*	*Nd*	*Pr*	*Sm*	*Tb*	*Tm*	*Yb*
**F**	10.01	6.95	9.05	5.50	5.86	4.42	7.07	6.64	4.34	5.58	18.20
**p**	$6.0*10^{-7}$	$3.0*10^{-5}$	$1.9*10^{-3}$	$3.0*10^{-4}$	$1.6*10^{-4}$	$1.4*10^{-3}$	$3.0*10^{-5}$	$5.0*10^{-5}$	$1.6*10^{-3}$	$2.0*10^{-4}$	$1.8*10^{-10}$

Table S13. Spanish commercial wines with their provenance, sample codification, vintage, color, grape variety and aging.

Protected designation of origin (PDO)	Sample codification	Vintage	Color	Grape variety	Aging
Alicante	*A1*	2014	Red	Cabernet Sauvignon, Tempranillo, Monastrell, Merlot	Crianza[&]
	A2	2016	Red	Shiraz	--
	A3	2016	White	Merseguere, Macabeo	--
	A4	2016	White	Moscatel, Chardonnay	--
	A5	2017	Red	Monastrell, Cabernet Sauvignon, Tempranillo	--
	A6	2017	White	Moscatel	--
	A7	2017	White	--	--
Bullas	*B1*	2010	Red	Monastrell	--
	B2	2014	Red	Monastrell, Syrah, Cabernet Sauvignon, Tempranillo	Young[*]
	B3	2015	Red	Monastrell	--
Campo de Borja	*CB1*	2015	Red	Garnacha, Tempranillo	Crianza[&]
	CB2	2017	Red	Garnacha	--
Jumilla	*J1*	2017	Red	Monastrell, Syrah	--
	J2	2017	Red	Monastrell, Tempranillo	Young[*]
Castilla la Mancha	*M1*	2013	Red	Tempranillo	Reserva[@]
	M2	2015	Red	Tempranillo	Crianza[&]
	M3	2015	Red	Tempranillo	Crianza[&]
	M4	2017	Red	Tempranillo	Young[*]
	M5	2017	Red	Tempranillo	--
Ribeiro	*R1*	2016	White	--	--
	R2	2017	White	Albariño	Young[*]
	R3		White	--	--

Ribera del Duero	*RD1*	2014	Red	Tempranillo	Crianza[&]
	RD2	2015	Red	Tempranillo	Crianza[&]
	RD3	2015	Red	Tempranillo	Roble[#]
	RD4	2015	Red	--	Crianza[&]
	RD5	2016	Red	Tempranillo	Roble[#]
	RD6	2016	Red	Tempranillo	Young[*]
Rioja	*RJ1*	2013	Red	Tempranillo, Garnacha	Reserva[@]
	RJ2	2013	Red	--	Reserva[@]
	RJ3	2015	Red	Tempranillo, Garnacha	Crianza[&]
	RJ4	2015	Red	--	Crianza[&]
	RJ5	2015	Red	Tempranillo	Crianza[&]
	RJ6	2016	Red	Tempranillo, Garnacha	Young[*]
	RJ7	2016	Red	Tempranillo	Young[*]
	RJ8	2017	Red	--	--
	RJ9	2017	Red	Tempranillo	Young[*]
	RJ10	2017	Red	Tempranillo	--
	RJ11	2017	White	Viura	--
	RJ12	2017	White	--	Young[*]
	RJ13	2017	White	--	--
	RJ14	2017	White	Viura	--
	RJ15		Red	Tempranillo	--
	RJ16		White	Viura, Malvasia	--
Rueda	*RU1*	2016	White	Verdejo	--
	RU2	2017	White	Verdejo	--

	RU3	2017	White	Verdejo	--
	RU4	2017	White	Verdejo	--
	RU5		White	--	--
Utiel-Requena	*U1*	2011	Red	--	Reserva@
	U2	2012	Red	Bobal, Tempranillo	Crianza&
	U3	2013	Red	Tempranillo	Reserva@
	U4	2014	Red	Bobal, Tempranillo	Reserva@
	U5	2015	Red	Tempranillo, Garnacha, Bobal	Crianza&
	U6	2015	Red	Tempranillo	Crianza&
	U7	2016	Red	Bobal, Tempranillo	--
Valdepeñas	*VP1*	2014	Red	Tempranillo	Crianza&
	VP2	2015	Red	Tempranillo	Crianza&
	VP3	2015	White	--	Crianza&
	VP4		Red	Tempranillo	--
Valencia	*V1*	2016	Red	Bobal, Shiraz	--
	V2	2016	White	Chardonnay, Sauvignon Blanc, Macabeo	--
	V3	2017	White	Viura, Sauvignon Blanc	--
	V4	2017	White	Viura, Sauvignon Blanc	--
	V5		White	Macabeo, Merseguera	Young*

--: Not provided; *Young: no aging process; #Roble: less than 6 months in oak barrels; &Crianza: minimum aging period of 24 months (18 months for white wine), at least 6 of which have been spend in oak barrels (12 months in La Rioja and Ribera del Duero regions); @Reserva: minimum aging period of 36 months (24 months for white wine), at least 12 of which are spent in oak barrels (6 for white wine).

Table S15. ICP-MS operating conditions.

**Sample introduction system (hTISIS)**	
Liquid flow rate/µL min^{-1}	30
Temperature/°C	150
Nebulizer gas flow rate/L min^{-1}	0.4
Ar HMI flow rate/L min^{-1}	0.56
**Plasma**	
Plasma gas flow rate/L min^{-1}	15.0
Auxiliary gas flow rate/L min^{-1}	1.0
RF Power/W	1600
**Collision cell**	
He flow rate/mL min^{-1}	4.3
OctP Bias/V	-18
Oct RF/V	200
Energy discrimination/V	3.0
**Acquisition parameters**	
Number of replicates	5
Integration time/s	0.3
Sweeps per replicate	100
**Measured ions**	
Analytes	$^{51}V^+$, $^{52}Cr^+$, $^{55}Mn^+$, $^{59}Co^+$, $^{60}Ni^+$, $^{111}Cd^+$, $^{137}Ba^+$, $^{139}La^+$, $^{141}Pr^+$, $^{146}Nd^+$, $^{147}Sm^+$, $^{153}Eu^+$, $^{157}Gd^+$, $^{159}Tb^+$, $^{163}Dy^+$, $^{165}Ho^+$, $^{166}Er^+$, $^{169}Tm^+$, $^{172}Yb^+$, $^{175}Lu^+$, $^{208}Pb^+$
Internal standards	$^{72}Ge^+$, $^{103}Rh^+$, $^{185}Re^+$
